# On the Inside

**DOI:** 10.1093/plphys/kiab104

**Published:** 2021-03-18

**Authors:** Peter V Minorsky

**Affiliations:** School of Health and Natural Sciences, Mercy College, Dobbs Ferry, New York, USA

## Heat shock protein promotes flowering under nonstressful conditions

Heat shock proteins (HSPs) are ubiquitously found throughout all kingdoms of life, and are typically made when cells are briefly exposed to temperatures above their normal growth temperature. HSPs serve as molecular chaperones to prevent the aggregation of misfolded proteins induced by stress and also to assist in the refolding of such proteins. HSP101, in particular, plays a key role in thermotolerance in plants. Transgenic Arabidopsis (*Arabidopsis thaliana*) plants that show reduced expression of *HSP101* are less tolerant to heat shock than wild-type plants, and *HSP101* mutants show inferior thermotolerance. Whether HSP101 functions in development under nonstress conditions, however, is not yet known. Based on the physiological, molecular, and genetic investigations, Qin et al. (pp. 407–420) now report that HSP101 plays a role in the control of flowering in Arabidopsis under nonstressful conditions. They report that the knockout and overexpression of *HSP101*, respectively, cause late and early flowering in Arabidopsis. HSP101 apparently regulates the expression of genes involved in each of the six known flowering pathways in this model species, most notably FLOWERING LOCUS C (FLC) and SHORT VEGETATIVE PHASE (SVP), which mostly repress flowering and are upregulated by HSP101. The late flowering phenotype of loss-of-*HSP101* mutants is suppressed by mutations of *FLC* and *SVP*. These results reveal a previously unknown function of HSPs under nonstressful conditions as a regulator of flowering time regulation.

## Phloem unloading in cucumber fruits

Phloem unloading is the process by which photoassimilates exit the sieve element/companion cell (SE/CC) complex in sink tissues, including fruits. In cucumber (*Cucumis sativus*) fruits, the phloem is unloaded via an apoplasmic pathway. Apoplasmic pathways for unloading require sugar transport carriers such as SWEET proteins to export sugars from the SE/CC complex. Different SWEET variants are specialized to use different sugars as substrates. In cucumber fruit, the dominant sugars unloaded from the phloem are glucose and fructose, although the major transported sugars are raffinose and stachyose. Stachyose and raffinose are likely hydrolyzed to sucrose by ɑ-galactosidase and then converted into hexoses by sucrose synthase or invertase. Li et al. (640–654) now report that a hexose transporter, CsSWEET7a, is highly expressed in cucumber sink tissues and is localized to the plasma membrane in CCs of the phloem. Downregulation of *CsSWEET7a* by RNA interference (RNAi) resulted in smaller fruit size along with reduced soluble sugar levels and reduced allocation of ^14^C-labeled carbon to sink tissues. *CsSWEET7a* overexpression lines showed an opposite phenotype. The authors also report that alkaline α-galactosidase and sucrose synthase were differentially regulated in parallel with *CsSWEET7a* expression. Immunohistochemical analysis demonstrated that ɑ-galactosidase was co-localized with CsSWEET7a in CCs, indicating cooperation between ɑ-galactosidase and CsSWEET7a in fruit phloem unloading. The apparent synergy between a SWEET transporter and soluble sugar hydrolases provides new insights into the molecular mechanisms underlying sugar transport during cucumber fruit development and suggests a possible approach toward improving fruit production in cucumber.

## An apoptosis-related protein involved in hybrid necrosis

Apoptosis and necrosis are two distinct types of cell death. A common apoptotic event in plants occurs when a pathogen attacks a plant, and the plant immune response initiates a programmed cell death (PCD)-like cell death called the hypersensitive response at the site of infection in order to limit the spread of the pathogen. The PCD response is triggered by the direct or indirect recognition of effectors from the invading pathogen by plant nucleotide-binding leucine-rich repeat (NLR) proteins. Compared to apoptosis, necrosis remains poorly understood in plants. Necrosis refers to cell death caused by external factors, such as injury or trauma, resulting in the unregulated degradation of cells and premature death. A special type of necrosis, called hybrid necrosis, is a significant problem encountered by plant breeders. Hybrid necrosis, a process characterized by the death of the whole hybrid plant, can occur in certain types of F_1_ hybrids. Hybrid necrosis often prevents the combination of desirable traits from different genotypes. The genes responsible for hybrid necrosis in *Arabidopsis thaliana* have been cloned, and include *DANGEROUS MIX* (*DM*) genes *DM1* and *DM2*, both of which encode NLR proteins. Hybrid necrosis is especially problematic for wheat (*Triticum aestivum*) breeders who, in attempting to induce heterosis in wheat, frequently encounter the problem of the premature death of certain F_1_s regardless of external factors. Jia et al. (pp. 483–497) have now identified a semi-dominant mutant allele that causes the necrotic death of wheat seedlings in the absence of any pathogen or external stimulus. Positional cloning of the lethal allele *mDES1* revealed that premature death via necrosis was caused by a point mutation in an NLR protein. *DES1* is an ortholog of *Sr35*, which recognizes a *Puccinia graminis* stem rust disease effector in wheat, but the *mDES1* allele apparently acquired a new function as a direct inducer of plant death. These findings shed light on the intersection of necrosis, apoptosis, and autoimmunity in plants.

## Ornithine decarboxylase and pollen rejection

Hybridization between related plant species or populations is prevented in nature by a variety of pre- and post-fertilization reproductive barriers. One form of reproductive barrier, unilateral incompatibility (UI), is unidirectional, in that the pollen of one species is rejected by pistils of another, but not vice versa. A correlation relating to UI, known as the “self-incompatible (SI) x self-compatible (SC) rule”, summarizes the general finding that the pollen of SC species or populations are typically incompatible on pistils of related SI species or populations. In the Solanaceae, SI involves a gametophytic S-RNase-based mechanism, wherein a polymorphic S-locus encodes highly variable pistil S-RNases that underlie the pollen incompatibility reaction. In the Solanaceae, UI occurs when the pollen lacks resistance to stylar S-RNases, but other, S-RNase-independent incompatibility mechanisms exist. Pistils of a wild tomato *Solanum pennellii* (SC), for example, lack S-RNase yet reject cultivated tomato (*Solanum lycopersicum*, SC) pollen. In this cross, UI results from low pollen expression of a *farnesyl pyrophosphate synthase* gene (*FPS2*) in *S. lycopersicum*. Using pollen from *fps2^−/−^* loss-of-function mutants in *S. pennellii*, Qin and Chetelat (pp. 452–468) have identified a pistil factor locus, *ui3.1*, required for *FPS2*-based pollen rejection. Thus locus maps to an interval containing 108 genes, including a cluster of *ornithine decarboxylase* (*ODC2*) genes. The *S. pennellii* genome contains *ODC2* genes and expresses 1034-fold higher *ODC2* transcript levels in pistils than *S. lycopersicum*, which contains a single gene. Pistils expressing wild-type *S. pennellii ODC2* genes reject *fps2* pollen, whereas *odc2* knockout mutants fail to do so. These results suggest that polyamines—the product of ODC activity—synthesized in the pistil contribute to S-RNase-independent pollen rejection. It is also demonstrated that *ODC2* genes interact genetically with *ui12.1*, a UI locus thought to encode two SI proteins, to strengthen pollen rejection, suggesting the *ODC2*-dependent pollen rejection mechanism may overlap with SI.

## The sphingolipidomes of various plant membranes

Various membrane lipid classes affect membrane parameters such as thickness, stability, permeability, curvature, fluidity, lateral diffusion, asymmetry, and interdigitation, thereby influencing the physiological role of the membrane during plant developmental processes and in stress responses. Three of the most common and abundant lipid families in plant membranes are: glycerophospholipids, sterols, and sphingolipids. In plants, complex sphingolipids are classified into four classes, ceramides (Cers), hydroxyceramides (hCERs), glucosylceramides, and glycosylinositolphosphoceramides (GIPCs), which constitute approximately 4%, 3%, 37%, and 56%, respectively, of the total sphingolipids extracted from Arabidopsis leaves. With the aim of describing the hydrophobic portion of sphingolipids, Carmona-Salazar et al. (pp. 624–640) isolated and quantified the sphingolipids from the microsomal (MIC), vacuolar (VM), plasma membrane (PM), and detergent-resistant membranes (DRMs) from Arabidopsis leaves. The four sphingolipid classes were determined to be present at different levels in each membrane: the VM was enriched in glucosylceramides, hydroxyceramides, and GIPCs; MICs in GIPCs and hCERs; PMs in GIPCs, in agreement with their key role in signal recognition and sensing; and DRMs in GIPCs, as expected from their function in nanodomain formation. While 84 sphingolipid species were identified in MICs, VMs, PMs, and DRMs, only 34 were selectively distributed in the 4 membrane types. Conversely, every membrane contained a different number of predominant species (11 in VMs, 6 in PMs, and 17 in DRMs). This study reveals that MICs, VMs, PMs, and DRMs contain the same set of sphingolipid species but every membrane source contains its own specific assortment based on the proportion of sphingolipid classes and on the predominance of individual species.

## Xylem network connectivity and embolism spread

Xylem vessels, when functional, deliver water from the roots to the plant canopy, where evaporation from the leaf creates a pressure gradient that drives xylem water transport. However, during severe drought conditions, xylem sap pressures may become so negative that embolisms form. Although these embolisms can spread from one water conducting cell to the next through pits in the cell walls, the three-dimensional complexity and scale of xylem networks and, thus, the functional implications of intervessel connections are not well understood. Wason et al. (pp. 392–406) have reconstructed the xylem networks of grapevine (*Vitis vinifera*) reconstructed by means of three-dimensional high-resolution X-ray micro-computed tomography images. Xylem network performance was then modeled to simulate loss of hydraulic conductivity under increasingly negative xylem sap pressures. The sensitivity of the xylem network to changes in key network parameters was also considered. Simulations showed that network organization imparted additional resistance to embolism spread beyond the air-seeding threshold of pit membranes. Xylem network vulnerability to embolism spread was most sensitive to pit membrane vulnerability and to variations in the number and location of vessels that were initially embolized. These results reveal that xylem network organization can increase stem resistance to embolism spread by 40% and challenge the notion that a single embolism can spread rapidly throughout an entire xylem network.

